# Arabidopsis inositol polyphosphate multikinase delays flowering time through mediating transcriptional activation of *FLOWERING LOCUS C*

**DOI:** 10.1093/jxb/erx397

**Published:** 2017-11-17

**Authors:** Sihong Sang, Yao Chen, Qiaofeng Yang, Peng Wang

**Affiliations:** State Key Laboratory of Hybrid Rice, College of Life Sciences, Wuhan University, China

**Keywords:** Arabidopsis, AtIPK2β, chromatin silencing, *FLC* expression, flowering time, FVE, transcriptional regulation

## Abstract

Timely flowering is critical for successful reproduction and seed yield in plants. A diverse range of regulators have been found to control flowering time in response to environmental and endogenous signals. Among these regulators, FLOWERING LOCUS C (FLC) acts as a central repressor of floral transition by blocking the expression of flowering integrator genes. Here, we report that Arabidopsis inositol polyphosphate multikinase (AtIPK2β) functions in flowering time control by mediating transcriptional regulation of *FLC* at the chromatin level. The *atipk2β* mutant flowers earlier, and AtIPK2β overexpressing plants exhibit late-flowering phenotypes. Quantitative reverse transcription-PCR (qRT-PCR) revealed that AtIPK2β promotes *FLC* expression. We performed chromatin immunoprecipitation-qPCR (ChIP-qPCR) assays and found that AtIPK2β binds to *FLC* chromatin. Further analysis showed that AtIPK2β interacts with FVE, a key repressor required for epigenetic silencing of *FLC*. qRT-PCR, ChIP-qPCR, and genetic analysis demonstrated that *AtIPK2β* is involved in FVE-mediated transcriptional regulation of *FLC* by repressing the accumulation of FVE on *FLC*. Moreover, we found that AtIPK2β associates with HDA6, an interaction partner of FVE mediating *FLC* chromatin silencing, and attenuates HDA6 accumulation at the *FLC* locus. Taken together, these findings suggest that AtIPK2β negatively regulates flowering time by blocking chromatin silencing of *FLC*.

## Teaser

Arabidopsis inositol polyphosphate multikinase (AtIPK2β) functions in the activation of *FLC* expression by blocking FVE-mediated chromatin silencing of *FLC*, delaying flowering time.

## Introduction

In Arabidopsis, timely flowering is ensured by an intricate regulatory network that has evolved in responde to environmental conditions and internal cues. The mainly genetic pathways are well defined, including photoperiod, vernalization, thermosensory, gibberellins (GA), age, and autonomous pathways (Blázquez *et al.*, 2003; Srikanth and Schmid, 2011; Wang, 2014). Among these pathways, the vernalization and autonomous pathways converge on *FLOWERING LOCUS C* (*FLC*), which encodes a MADS-box transcription factor that blocks flowering by transcriptional repression of floral integrator genes including *FLOWERING LOCUS T* (*FT*) and *SUPPRESSOR OF OVEREXPRESSION OF CO 1* (*SOC1*) (Sheldon *et al.*, 2000; Simpson, 2004; Parcy, 2005; Lee and Lee, 2010). *FLC* acts as a floral repressor by antagonizing the activity of pathways promoting flowering in a dose-dependent manner. Its expression is regulated at the transcriptional and post-transcriptional levels by diverse factors (Amasino, 2010; He, 2012). Although previous investigations have revealed different regulators in the control of *FLC* expression, more studies are needed to provide better understanding of the complex regulation in *FLC*-mediated flowering.

Chromatin modification plays a crucial role in transcriptional regulation of *FLC*. Active modifications at *FLC* chromatin, such as histone H3 acetylation (H3Ac), lysine-4 methylation (H3K4me2/H3K4me3), lysine-36 methylation (H3K36me2/H3K36me3), and histone H2B monoubiquitination (H2Bub1), induce gene expression, whereas repressive modifications, such as histone deacetylation, histone lysine-9 methylation, and histone H3 lysine-27 trimethylation (H3K27me3), result in silencing of *FLC* (Choi *et al.*, 2011; He, 2012). Histone modification at the *FLC* locus is mediated by various multiprotein complexes, such as the Polycomb Repressive Complex 2 (PRC2)-like complex and histone deacetylase complexes, which silence *FLC* by depositing the repressive H3K27me3 mark or removing the active H3Ac mark at the *FLC* locus, and the FRIGIDA (FRI) complex, which activates *FLC* expression by accumulating the active H3Ac, H3K4me3, H3K36me2, and H3K36me3 marks on the chromatin (De Lucia *et al.*, 2008; Ko *et al.*, 2010; Choi *et al.*, 2011; He, 2012). A number of proteins functioning in the chromatin-regulating complexes have been identified in both the vernalization and autonomous pathways to mediate histone modifications at *FLC* (He, 2012; Simpson, 2004).

Among these proteins, FVE, a key component of the autonomous flowering pathway containing histone-binding and WD40-repeat motifs, is required for silencing *FLC* by mediating chromatin modifications on the gene locus (Jeon and Kim, 2011; Pazhouhandeh *et al.*, 2011). FVE is homologous to the mammalian retinoblastoma-associated proteins RbAp46/RbAp48 and is also known as Arabidopsis MULTICOPI SUPPRESSOR OF IRA1 4 (AtMSI4), which belongs to the MSI1-like protein family that is conserved in yeast and plants (Ausín *et al.*, 2004; Hennig *et al.*, 2005; Gu *et al.*, 2011). FVE associates with CULLIN4-Damaged DNA Binding Protein1 (CUL4-DDB1) and PRC2-like complexes, and is required for effective H3K27 trimethylation on both *FLC* and *FT* chromatin (Pazhouhandeh *et al.*, 2011). Additionally, FVE interacts with Histone Deacetylase 6 (HDA6) to mediate histone deacetylation and DNA methylation, resulting in silencing of the target genes (Gu *et al.*, 2011; Xu *et al.*, 2013*b*). The complex formed by FVE, HDA6, and FLOWERING LOCUS D (FLD) represses *FLC* expression by reducing accumulation of the active H3K4me3 and H3Ac marks and promoting deposition of the repressive H3K27me3 mark (Yu *et al.*, 2016). Moreover, the interaction between FVE and HIGH EXPRESSION OF OSMOTICALLY RESPONSIVE GENE1 (HOS1), an E3 ligase that mediates protein degradation, blocks the function of HDA6 by inhibiting the binding of HDA6 to *FLC* chromatin in response to short-term cold stress, resulting in delayed floral transition (Jung *et al.*, 2013). Collectively, these studies have shown pleiotropic functions of FVE in the regulation of *FLC* expression at the chromatin level by interacting with different proteins.

Inositol polyphosphate multikinases (IPMKs) play important roles in inositol phosphate metabolism and signal transduction. The conserved catalytic activities of IPMKs are phosphorylating inositol 1,4,5-triphosphate (IP_3_) to generate inositol 1,4,5,6-tetrakisphosphate (IP_4_) and inositol 1,3,4,5,6-pentakisphosphate (IP_5_) (Chang and Majerus, 2006; Resnick and Saiardi, 2008). IPMKs and their products are involved in the regulation of gene expression. IP_4_ and IP_5_ produced by yeast IPMK (also known as IPK2) act as stimulators in modulating the activities of chromatin-remodeling complexes, such as INO80 and SWI/SNF, resulting in activation of target genes (El Alami *et al.*, 2003; Steger *et al.*, 2003). IP_4_ also functions as a key component of mammalian class I histone deacetylase (HDAC) complexes (Millard *et al.*, 2013). Independent of their catalytic activities, IPMKs function as co-activators in transcriptional regulation. The yeast IPMK/IPK2 interacts with a MADS-box protein, Mcm1, and stabilizes the complex Mcm1-ArgR, which recognizes the specific DNA sequences termed the ‘arginine box’ in the promoter region and activates the target gene (El Bakkoury *et al.*, 2000; Bosch and Saiardi, 2012). In mammalian cells, IPMK interacts with histone acetyltransferase CBP and histone acetyltranferase complex p53-p300 to mediate transcriptional activation (Xu *et al.*, 2013*a*; Xu and Snyder, 2013; Kim *et al.*, 2016). Moreover, a class II HDAC identified in *Tetrahymena* contains an IPMK domain homologous to IPK2 (Smith *et al.*, 2008). AtIPK2β, an IPMK identified in Arabidopsis, exhibits conserved 6-/3-kinase activity and is homologous to yeast IPK2, as expressing AtIPK2β in the *IPK2* deletion yeast partially compensates its growth defects (Stevenson-Paulik *et al.*, 2002; Xia *et al.*, 2003; Bosch and Saiardi, 2012). A recent study showed that the kinase activity of AtIPK2β is essential for its function in plant sexual reproduction, including in pollen development, pollen tube guidance, and embryogenesis (Zhan *et al.*, 2015). Several investigations have also suggested a potential role of AtIPK2β in transcriptional regulation. AtIPK2β is enriched in the nucleus and regulates the expression of auxin-responsive genes (Xia *et al.*, 2003; Zhang *et al.*, 2007). Moreover, ectopic expression of *AtIPK2β* in tobacco enhances the tolerance of transgenic plants to abiotic stresses through promoting the transcription of stress-responsive genes (Yang *et al.*, 2008). However, the molecular mechanism through which AtIPK2β mediates transcriptional regulation is still unknown.

In this study, we report the function of AtIPK2β in flowering time control via mediating the transcriptional regulation of *FLC*. AtIPK2β acts as a negative regulator of flowering time by promoting the expression of *FLC*. Chromatin immunoprecipitation-quantitative PCR (ChIP-qPCR) analysis revealed that AtIPK2β mediates transcriptional regulation of *FLC* via binding to *FLC* chromatin. In addition, AtIPK2β interacts with FVE and represses the accumulation of FVE at the *FLC* locus. Gene expression, genetic, and ChIP-qPCR assays confirmed that AtIPK2β is involved in FVE-mediated transcriptional regulation of *FLC* by affecting chromatin modifications including histone H3K27 trimethylation and H3 deacetylation at *FLC*. Moreover, AtIPK2β associates with HDA6, an interaction partner of FVE, and attenuates the accumulation of HDA6 on *FLC* chromatin. In general, these results suggest that AtIPK2β functions as a repressor in flowering time control through promoting *FLC* expression at the chromatin level.

## Materials and methods

### Plant materials

All the plants used in this study were *Arabidopsis thaliana* Columbia type (Col-0) background. The *atipk2β-1* (SALK_025091) and *atipk2β-2* (SALK_104995) mutant lines were described previously by Zhang *et al.* (2007) and Zhan *et al.* (2015). The *flc-6* null mutant, obtained from the Arabidopsis Biological Resource Center, was originally described by Bouveret *et al.* (2006). The *fve* T-DNA line (SALK_013789) was provided by Dr Ligeng Ma, and genotyping PCR was performed for identifying the T-DNA insertion. Double mutants of *flc/atipk2β* and *fve/atipk2β* were generated by crossing the *fve* and *flc-6* lines, respectively, with *atipk2β-2.* The homozygous double mutants of F3 progenies were selected by genotyping PCR and used in this study. The primers used in identification of the mutants, with detailed sequences, are listed in Supplementary Table S1 at *JXB* online.

The complementary lines of the *atipk2β* mutant were generated by introducing a construct encoding an AtIPK2β-Green Fluorescent Protein (GFP) fusion protein under the control of the native promoter into *atipk2β-2*, as described by Zhan *et al.* (2015). For overexpression transgenic lines, the *AtIPK2β* or *FVE* coding sequence was inserted into the pCAMBIA1302 vector after the CaMV 35S promoter region and followed by the coding sequence of GFP. Col-0 and the *atipk2β-2* mutant plants were agro-transformed with the resulting vectors using the floral dip method (Clough and Bent, 1998), and T3 generation plants selected with hygromycin B were used in further analyses. *HDA6-MYC/atipk2β* overexpression plants were generated by crossing the *atipk2β* mutant with a *HDA6-MYC* transgenic line, which is in the Col-0 background and was provided by Dr Keqiang Wu (Liu *et al.*, 2012). The F3 progenies of *HDA6-MYC/atipk2β* were identified and used in ChIP-qPCR assays.

### Flowering time analysis

Plants were grown on soil in a controlled culture room at 22 °C with cold fluorescent light (100 μmol m^−2^ s^−1^) under long-day (LD; 16 h light) or short-day (SD; 8 h light) photoperiods. Seeds were stratified for 4–5 days at 4 °C on Murashige and Skoog (MS) agar plates before being transferred on to soil. Flowering time was measured by counting the number of rosette leaves and the number of days at bolting after germination. At least 10 plants were counted and averaged for each genotype.

### Gene expression analysis

For analyzing gene expression levels, seeds were sterilized with ethanol and then stratified for 4–5 days at 4 °C on solid MS agar plates. The plates were kept in a controlled culture room at 23 °C with cold fluorescent light (100 μmol m^−2^ s^−1^) under the LD or SD photoperiod for 15 to 20 days before being harvested. RNA was extracted using a TRIzol (Molecular Research Center) method and treated with DNase I (Promega) before being transcribed into cDNA by M-MLV Reverse Transcriptase (Promega). Quantitative reverse transcription-PCR (qRT-PCR) was performed with gene-specific primers (listed in Supplementary Table S1) on an ABI Step-one Plus real-time PCR system using SYBR Green Realtime PCR Master Mix (TOYOBO). The quantification results were normalized to two internal reference genes (*ACT2* and *UBQ10*) and then compared with the wild-type (WT) control plants. Data are presented as means±SD of at least two independent repeats with similar results.

### Yeast two-hybrid assays

The pGBKT7 and pGADT7 vectors of the BD Matchmaker system (Clontech) and the yeast strain AH109 were used for yeast two-hybrid assays according to the manufacturer’s instructions. The full-length coding sequence of *FVE* or *HDA6* was subcloned into pGADT7, and AtIPK2β was inserted into pGBKT7. The final constructs were transformed into yeast strain AH109. Transformants were spotted on to plates of selective medium without Leu and Trp (DDO), and further on medium without Leu, Trp, His, and Ade (QDO). Plates were incubated at 30 °C for 3–5 days. The filter β-galactosidase assays were conducted according to the system protocol (Clontech).

### Bimolecular fluorescence complementation assays

Arabidopsis protoplasts from rosette leaves of plants grown for 3 weeks under the LD photoperiod were isolated as described by Yoo *et al.* (2007). The coding sequence of *AtIPK2β* or *FVE* was inserted between the CaMV 35S promoter and terminator regions and fused to the sequences encoding the N-terminal fragment or C-terminal region of Yellow Fluorescent Protein (YFP) to form the *YN-AtIPK2β* or *YC-FVE* fusion, respectively. The DNA fragments including the CaMV 35S promoter, coding sequence of *YN-AtIPK2β* or *YC-FVE* fusion, and the terminator regions were amplified by PCR and transformed into the protoplasts according to the method described previously by Lu *et al.* (2013). An irrelevant YC-SnRK2.6 fusion employed for testing the efficiency of the bimolecular fluorescence complementation (BiFC) system by Lu *et al.* (2013) was used as a negative control, as well as empty YN or YC constructs. Fluorescence of YFP was visualized under an Olympus FV 1000 confocal microscope within 18 hours after transformation.

### Immunoblot assays

Arabidopsis rosette leaves of indicated genotypes grown on MS agar plates for 15 days under the LD photoperiod were homogenized in extraction buffer containing 50 mM Tris-HCl (ph 7.5), 100 mM NaCl, 10% glycerol, 0.1% Nonidet P-40, 1 mM phenylmethylsulfonyl fluoride, and 1× complete protease inhibitor (Roche). Total protein was extracted by centrifugation at 4 °C. The protein concentration was determined by using a Bradford assay kit (BCA, Thermo Scientific). Western blot was performed by blotting protein samples on polyvinylidene fluoride membranes (Millipore) after being boiled and run on SDS-PAGE gels. Different primary antibodies were used for probing the blots. A polyclonal AtIPK2β antibody was raised in rabbits as described by Yang *et al.* (2008).

### Co-immunoprecipitation assays

Co-immunoprecipitation (Co-IP) assays were performed as described previously (Chen *et al.*, 2010). Approximately 1–2 mg total proteins extracted from leaves of transgenic Arabidopsis were incubated with an anti- GFP antibody or anti-AtIPK2β antibody overnight at 4 °C, and then Protein A magnetic beads (NEB) were added and incubated for another 2 hours. Subsequently, the beads were washed according to the immunoprecipitation protocol (NEB) and the eluted precipitates were analyzed by western blot using anti-MYC, anti-GFP, or anti-AtIPK2β antibodies.

### Chromatin immunoprecipitation-quantitative PCR assays

ChIP experiments were performed according to the method described previously (Saleh *et al.*, 2008). Plants 10–15 days old grown on MS agar plates under a LD photoperiod were collected for chromatin extraction. Antibodies used in ChIP assays included anti-GFP, anti-AtIPK2β, anti-MYC, anti-trimethylated-H3 (Lys 27) (Millipore, 07-449), and anti-acetyl-H3 (Abcam, ab47915). The procedure and data calculation of ChIP-qPCR were conducted as described previously (Yamaguchi *et al.*, 2014). DNA fragments of *ACTIN2* (*ACTIN*) and *FUSCA3* (*FUSCA*) were used for normalization (*ACTIN* for H3Ac and *FUSCA* for H3K27me3) in the ChIP-qPCR assays to detect enrichments of H3Ac and H3K27md3 marks on *FLC* as described previously (Gu *et al.*, 2011; Pazhouhandeh *et al.*, 2011). *ACTIN* was also used for normalization in the ChIP-qPCR assays conducted to examine the accumulation of AtIPK2β, AtIPK2β-GFP, FVE-GFP, and HDA6-MYC on *FLC*, as the indicated proteins were not found to be enriched in this region at the *ACTIN2* locus. Primers used in the ChIP-qPCR and their detailed sequences are listed in Supplementary Table S2.

## Results

### AtIPK2β delays flowering time

The *AtIPK2β* gene encodes a conserved IPMK in higher plants with diverse functions in growth and development, including abiotic stress response, axillary branching, and sexual reproduction (Stevenson-Paulik *et al.*, 2002; Xia *et al.*, 2003; Zhang *et al.*, 2007; Yang *et al.*, 2008; Zhan *et al.*, 2015). However, it is unclear whether AtIPK2β plays a role in flowering time regulation. We explored the possible function of *AtIPK2β* in the regulation of flowering time using the *atipk2β* mutant and *AtIPK2β* overexpression plants. Two *atipk2β* RNA-null mutant lines, *atipk2β-1* and *atipk2β-2*, which were described previously (Zhang *et al.*, 2007), were identified again by detecting the expression of *AtIPK2β* (Supplementary Fig. S1A, B). We observed that both of the *atipk2β* mutant lines flowered earlier and had reduced numbers of rosette leaves at bolting compared with WT plants under LD growth conditions (Supplementary Fig. S1C). The *atipk2β* mutant we used in the following studies was the *atipk2β-2* line. We next confirmed that the early flowering of the *atipk2β* mutant was due to loss of *AtIPK2β* expression by performing genetic complementation experiments. An *AtIPK2β-GFP* fusion construct under the control of the native promoter was introduced into the *atipk2β* mutant; both of the complemented lines (Com1 and Com2) exhibited similar or slightly delayed flowering compared with WT (Supplementary Fig. S1C).

**Fig. 1.**
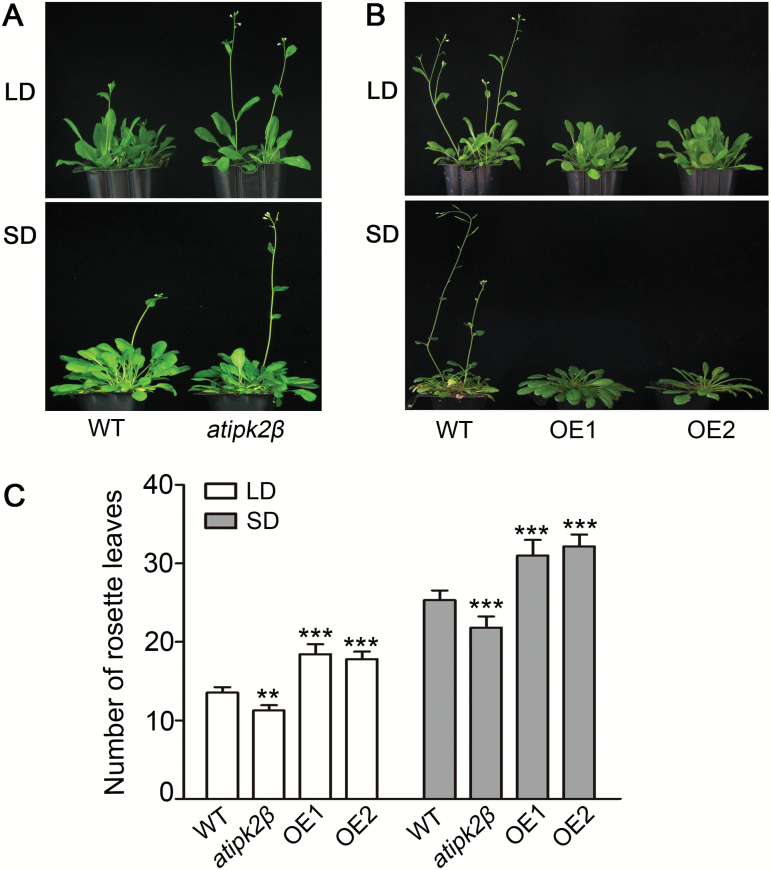
AtIPK2β delays flowering time. (A) Flowering phenotype of wild-type (WT) and *atipk2β* mutant plants grown under long-day (LD) and short-day (SD) photoperiods. (B) Flowering phenotypes of WT and *AtIPK2β* overexpressing lines grown under LD and SD conditions. Two lines of transgenic plants (OE1 and OE2) are shown. (C) Number of rosette leaves of WT, *atipk2β*, and *AtIPK2β* overexpressing (OE1 and OE2) plants at bolting. Rosette leaf numbers of 10–15 plants at flowering were counted and averaged for each genotype. Each value for the *atipk2β* mutant and overexpressing lines was compared with WT separately. Statistically significant differences were determined by one-way ANOVA with Tukey’s multiple comparison test (**P<0.01, ***P<0.001). (This figure is available in colour at *JXB* online.)

As the *atipk2β* mutant plants flowered earlier under both LD and SD conditions (Fig. 1A, C), we further confirmed the function of *AtIPK2β* by analyzing the flowering phenotype of the *AtIPK2β* overexpression lines generated by overexpressing AtIPK2β-GFP in the *atipk2β* mutant background. Two of the *AtIPK2β* overexpression lines (named OE1 and OE2) were used for further analysis. The *AtIPK2β* overexpression lines exhibited delayed flowering (Fig. 1B) and produced significantly more rosette leaves at bolting under both LD and SD photoperiods (Fig. 1C). These results suggested that *AtIPK2β* acts as a negative regulator of flowering time independent of photoperiod.

### AtIPK2β affects the expression of flowering-related genes

To investigate the possible molecular mechanism by which AtIPK2β regulates flowering time, four genes, *CONSTANS* (*CO*), *FLC*, *FT*, and *SOC1*, which encode key flowering-time regulators and floral integrators, were analyzed in the *atipk2β* mutant and *AtIPK2β* overexpression plants grown under both LD and SD conditions. The *AtIPK2β* overexpression line (OE1) exhibiting a late-flowering phenotype (Fig. 1B, C) was used in the gene expression analysis.

**Fig. 2.**
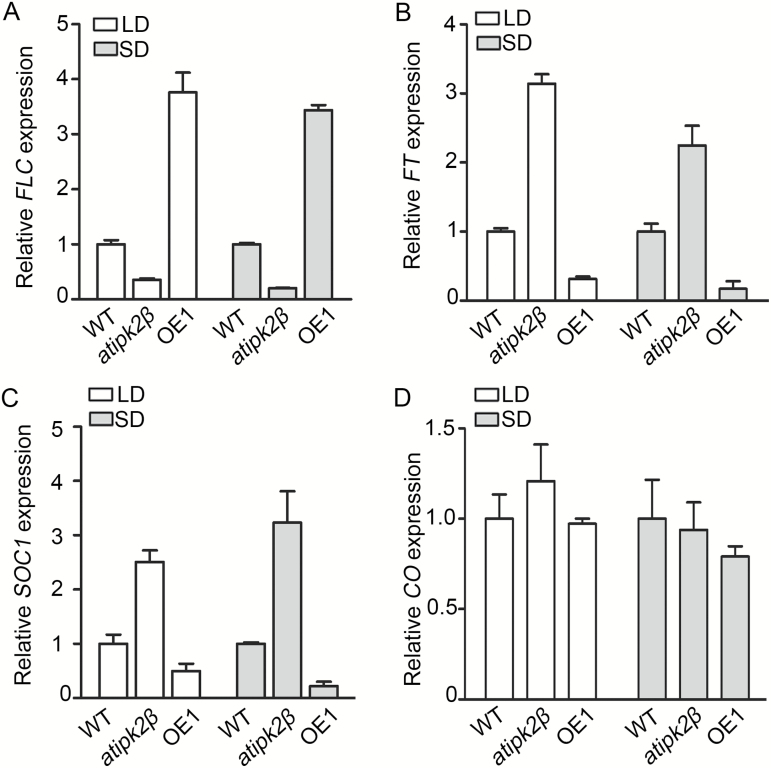
Expression analysis of flowering-related genes. Relative expression levels of *FLC* (A), *FT* (B), *SOC1* (C), and *CO* (D) in wild-type (WT), *atipk2β* mutant, and *AtIPK2β* overexpressing line (OE1) plants under long-day (LD) and short-day (SD) growth conditions. Plants of the indicated genotypes were grown under LD and SD photoperiods for 15 days and harvested at zeitgeber time (ZT) 7. The relative expression level of each gene was determined by qRT-PCR, followed by normalizing to *ACT2* and *UBQ10.* Data presented are the mean±SD of two biological replicates.

*FLC*, which encodes a key repressor of flowering, is transcriptionally regulated by multiple signaling pathways and factors (Srikanth and Schmid, 2011). To explore whether AtIPK2β regulates flowering by affecting the transcription of *FLC*, we examined the expression level of *FLC* in the *atipk2β* mutant and the *AtIPK2β* overexpression line OE1. This analysis showed that the level of *FLC* transcript decreased significantly in the *atipk2β* mutant and increased in OE1 compared with WT plants under both LD and SD photoperiods (Fig. 2A). Signaling pathways controlling flowering time converge on integrator genes including *FT* and *SOC1*, both of which function as floral activators and are repressed by FLC directly at the transcriptional level (Helliwell *et al.*, 2006; Lee and Lee, 2010; Srikanth and Schmid, 2011). We examined the expression levels of *FT* under both LD and SD growth conditions. As Fig. 2B shows, the transcription level of *FT* increased in the *atipk2β* mutant and decreased in *AtIPK2β* overexpressing plants, correlating inversely with the expression levels of *FLC*. Except when being repressed by FLC, *SOC1* is transcriptionally activated by FT directly (Lee and Lee, 2010). Similar to *FT*, the expression of *SOC1* was promoted in the *atipk2β* mutant and repressed in OE1 under both LD and SD photoperiods (Fig. 2C). These results indicate that *AtIPK2β* promotes *FLC* expression and thereby represses transcription of *FT* and *SOC1*.

We also examined the expression level of *CO*, which encodes a transcriptional activator of *FT* that responds to photoperiod (Suárez-López *et al.*, 2001). As *AtIPK2β* regulates flowering time in both LD and SD conditions, it may not be involved in the photoperiod pathway. In support of this hypothesis, the transcription level of *CO* was not affected in either *atipk2β* mutant or *AtIPK2β* overexpressing plants (Fig. 2D).

### *AtIPK2β* interacts genetically with *FLC*

**Fig. 3.**
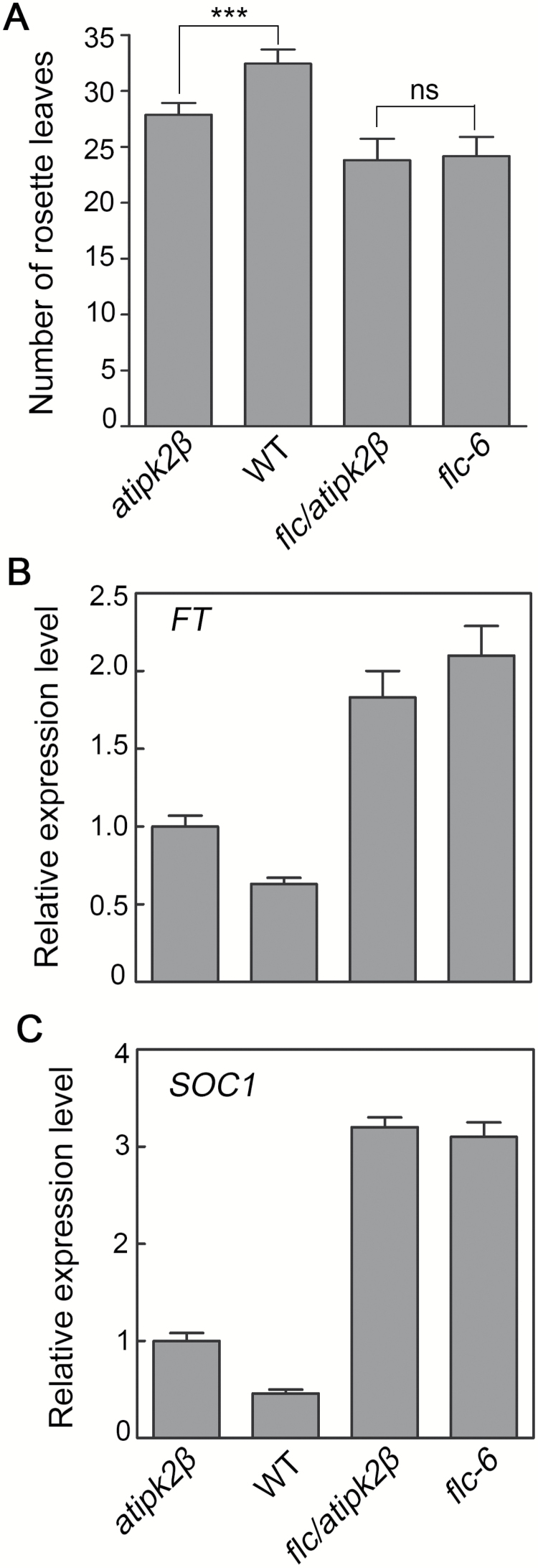
*AtIPK2β* interacts genetically with *FLC*. (A) Flowering time analysis of *atipk2β*, wild-type (WT), *flc/atipk2β*, and *flc-6* plants grown under short-day conditions. Numbers of rosette leaves of 10–15 plants at bolting were counted and averaged. Data are presented as means±SD. Data for WT were compared with *atipk2β*, and *flc-6* with *flc/atipk2β*, using one-way ANOVA with Tukey’s multiple comparison test (***P<0.001; ns, not significant). (B, C) Expression level of *FT* (B) and *SOC1* (C) in WT and mutant lines. 20-day-old plants grown under SD conditions were collected at zeitgeber time (ZT) 5 for detecting gene expression. Data presented are the means±SD of two biological repeats.

We further confirmed that *AtIPK2β* regulates *FLC* expression to control flowering time by investigating the genetic interaction between *AtIPK2β* and *FLC*. The double mutant *flc/atipk2β* was produced by crossing the *atipk2β* mutant with the *flc-6* null mutant (Bouveret *et al.*, 2006). As the early-flowering phenotype of the *flc-6* mutant under SD photoperiods is more significant than that under LD conditions (Steinbach and Hennig, 2014; Tsuchiya and Eulgem, 2010), we analyzed the flowering phenotypes of WT and *atipk2β*, *flc-6*, and *flc/atipk2β* mutants grown under SD conditions (Fig. 3A). All of the mutant plants flowered earlier than WT. Moreover, the *flc/atipk2β* and *flc-6* mutant lines exhibited a similar flowering time, suggesting that the expression of *AtIPK2β* in *flc-6* is unable to rescue the early-flowering phenotype of *flc/atipk2β*. We further examined the expression levels of *FT* and *SOC1* in the same mutant lines. Although expression of *AtIPK2β* in WT resulted in decreased transcription of *FT* and *SOC1* compared with the levels in *atipk2β, flc-6* exhibited similar expression levels of the two genes as *flc/atipk2β* (Fig. 3B, C), demonstrating that the flowering phenotype of the double mutant is due to the loss of *FLC.* These results indicated that the functions of *AtIPK2β* in regulating flowering time may require *FLC*.

### AtIPK2β binds to *FLC* chromatin in Arabidopsis

**Fig. 4.**
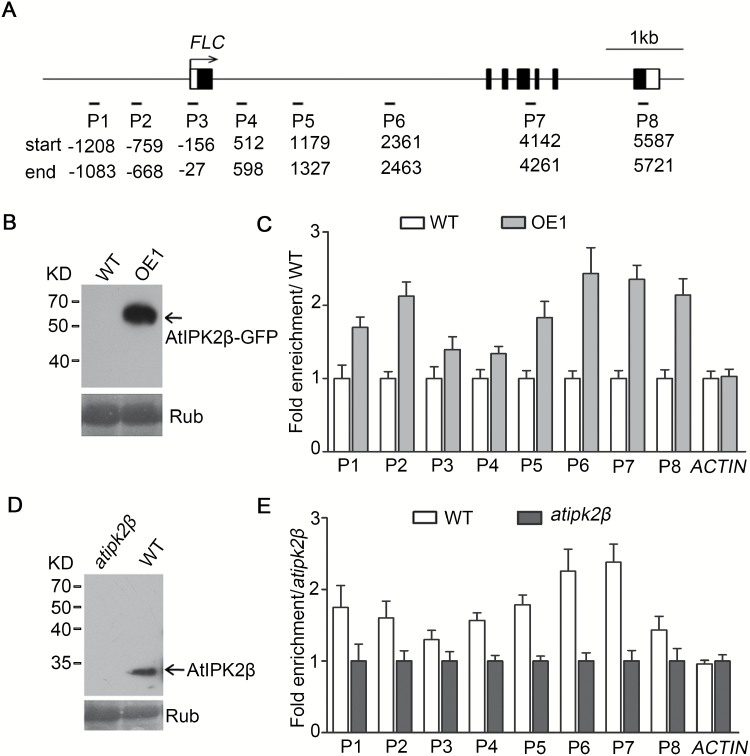
Binding of AtIPK2β to *FLC* chromatin in plants. (A) Schematic diagram of the *FLC* gene. The sequence regions marked P1–P8 indicate the eight regions examined in the ChIP assays, and the numbers below indicate residue positions relative to the ATG start codon. The black boxes represent exons and lines between them represent introns. White boxes denote the 5ʹ and 3ʹ untranslated regions. The arrow indicates the transcription start site of *FLC*. (B) Immunodetection of AtIPK2β-GFP fusion protein (arrow) with anti-GFP antibody in OE1 plants. The large subunit of ribulose 1,5-bisphosphate carboxylase (Rub) stained with Ponceau S is shown as a loading control in the lower panel. The position and size of standard protein markers are indicated to the left. (C) AtIPK2β binds to *FLC* chromatin in *AtIPK2β* overexpressing plants. 15-day-old plants of the OE1 line overexpressing *AtIPK2β-GFP* grown under long-day (LD) conditions were collected and an anti-GFP antibody was used for chromatin immunoprecipitation (ChIP). An *ACTIN2* DNA fragment (*ACTIN*) was used for normalizing the amount of precipitated DNA fragments quantified by qPCR. Fold enrichment levels of AtIPK2β-GFP in transgenic plants relative to the negative control [wild-type (WT)] are shown as the mean±SD of two biological repeats. (D) Immunodetection of AtIPK2β protein (arrow) with anti-AtIPK2β antibody in WT and *atipk2β* mutant plants. The positions and size of standard protein markers are indicated to the left. (E) Binding of AtIPK2β to the *FLC* locus in WT plants. ChIP-qPCR assays were performed using WT and the *atipk2β* mutant with an anti-AtIPK2β specific antibody, and the precipitated DNA fragments were quantified and then normalized to *ACTIN*. Plants were grown under LD conditions for 15 days and the *atipk2β* mutant was used as negative control. Fold enrichment levels of AtIPK2β at the *FLC* in WT relative to the *atipk2β* mutant are shown as the means±SD of two independent experiments.

Next, we performed ChIP-qPCR assays to investigate the molecular mechanism by which AtIPK2β regulates the transcription of *FLC*. Eight sequence regions within the *FLC* locus (P1–P8; Fig. 4A) were examined by ChIP-qPCR. The OE1 line overexpressing the *AtIPK2β-GFP* fusion construct and an anti-GFP antibody were used for ChIP. A band of AtIPK2β-GFP of the expected size (61 kDa) was detected in extracts from the transgenic plants (Fig. 4B). Chromatins extracted from OE1 were immunoprecipitated with anti-GFP antibody and the precipitated DNA fragments after ChIP were quantified by qPCR. ChIP-qPCR assays revealed that AtIPK2β binds to *FLC* chromatin; among the regions of FLC examined, the enrichment level of AtIPK2β was higher in sequence regions P2, P6, and P7 (Fig. 4C). We further confirmed the enrichment of AtIPK2β at the *FLC* locus using an anti-AtIPK2β antibody in WT plants, because the ectopic expression of GFP-tagged AtIPK2β may cause potential artifacts. The specificity of the antibody was verified by performing immunological analysis using WT and the *atipk2β* mutant. Western blot analysis showed that a band of AtIPK2β of the expected size (33 kDa) was detected with anti-AtIPK2β antibody in protein extracts from WT plants, but no such band was detected in the extracts from the *atipk2β* mutant (Fig. 4D). The ChIP-qPCR results showed that AtIPK2β binds to *FLC* chromatin and the enrichment levels were higher in the P6 and P7 regions (Fig. 4E). These data suggest a possible regulatory mechanism through which AtIPK2β promotes *FLC* expression by mediating chromatin regulation of *FLC.*

### AtIPK2β interacts with FVE *in vivo*

We further investigated how AtIPK2β regulates the transcription of *FLC*. IPMKs function in transcriptional regulation by binding to the other regulators or chromatin modifiers (El Bakkoury *et al.*, 2000; Kim *et al.*, 2016). It is possible that AtIPK2β also mediates the activation of *FLC* expression through a similar mechanism. Potential interaction proteins of AtIPK2β were identified by affinity purification and mass spectrometry (unpublished data) performed as described previously (Wang *et al.*, 2017), and one peptide corresponding to FVE was identified (Supplementary Fig. S2).

**Fig. 5.**
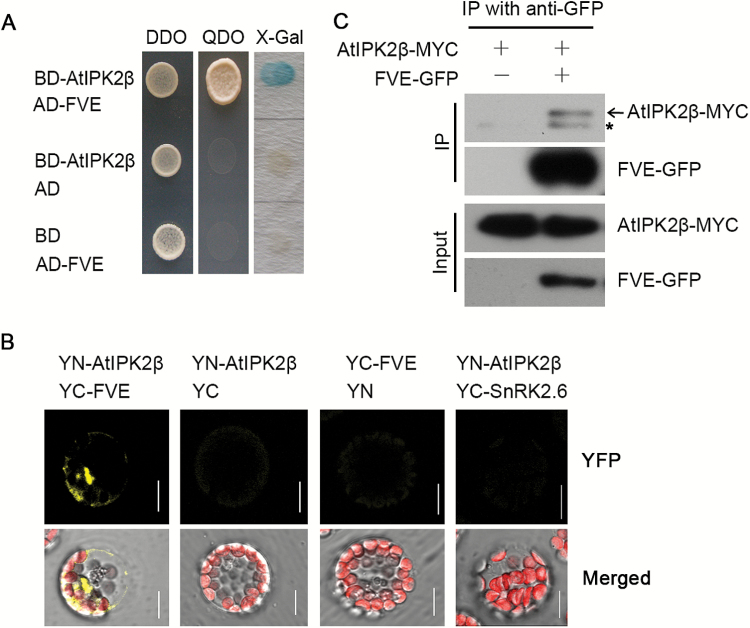
AtIPK2β interacts with FVE in yeast and plants. (A) Interaction of AtIPK2β with FVE in yeast. Yeast two-hybrid assays were used for testing the interaction. Yeast cells were streaked on the selective media DDO (SD/-Leu/-Trp) and QDO (SD/-Leu/-Trp/-His/-Ade). X-Gal indicates filter β-galactosidase assays. (B) BiFC assays of the interaction between AtIPK2β and FVE in Arabidopsis protoplasts. AtIPK2β and FVE were fused to the YFP N-terminal region (YN) and C-terminal region (YC) to form YN-AtIPK2β and YC-FVE, respectively. Empty YN and YC constructs or an irrelevant YC-SnRK2.6 fusion were used as negative controls. Confocal images of YFP, autofluorescence, and bright-field detections are merged and shown in the lower panels, and images of YFP are shown in the upper panels. Bars=10 μm. (C) AtIPK2β interacts with FVE in transgenic Arabidopsis. Co-immunoprecipitation (Co-IP) assays were conducted by extracting total proteins from transgenic plants co-expressing AtIPK2β-MYC and FVE-GFP fusions, which were then immunoprecipitated with an anti-GFP antibody. The precipitates were detected by western blot with an anti-MYC antibody. An *AtIPK2β-MYC* overexpressign line was used as the negative control. The asterisk indicates a non-specific IgG band. (This figure is available in colour at *JXB* online.)

FVE is a key regulator in the autonomous flowering pathway, which inhibits the expression of *FLC* by mediating histone modification on *FLC* chromatin (Gu *et al.*, 2011; Pazhouhandeh *et al.*, 2011). The potential interaction between FVE and AtIPK2β may explain how AtIPK2β regulates *FLC* expression. We first confirmed the interaction between AtIPK2β and FVE in yeast by performing yeast two-hybrid assays (Fig. 5A). BiFC assays were conducted to test whether AtIPK2β interacts with FVE in plants. The N-terminal fragment of YFP was fused to AtIPK2β to form YN-AtIPK2β, and the C-terminal half of YFP was fused to FVE to form YC-FVE. YN-AtIPK2β and YC-FVE were simultaneously expressed in Arabidopsis protoplasts and YFP fluorescence was observed (Fig. 5B), reflecting the physical association of AtIPK2β and FVE *in vivo*. Co-IP assays were conducted to further determine the interaction using transgenic plants co-expressing AtIPK2β-MYC and FVE-GFP fusion proteins; transgenic plants overexpressing *AtIPK2β-MYC* in the WT background was used as a negative control. The FVE-GFP fusion and the interacting proteins were co-immunoprecipitated by an anti-GFP antibody from the total proteins of *FVE-GFP/AtIPK2β-MYC* transgenic plants, and the precipitates were analyzed by western blot using an anti-MYC antibody. The band of AtIPK2β-MYC was detected in the protein extracts from the *FVE-GFP/AtIPK2β-MYC* transgenic plants, but not in the extracts from the negative control (Fig. 5C). Taken together, these results evidence the interaction between AtIPK2β and FVE, indicating a functional link between the two proteins.

### Binding of FVE to *FLC* chromatin is repressed by AtIPK2β

**Fig. 6.**
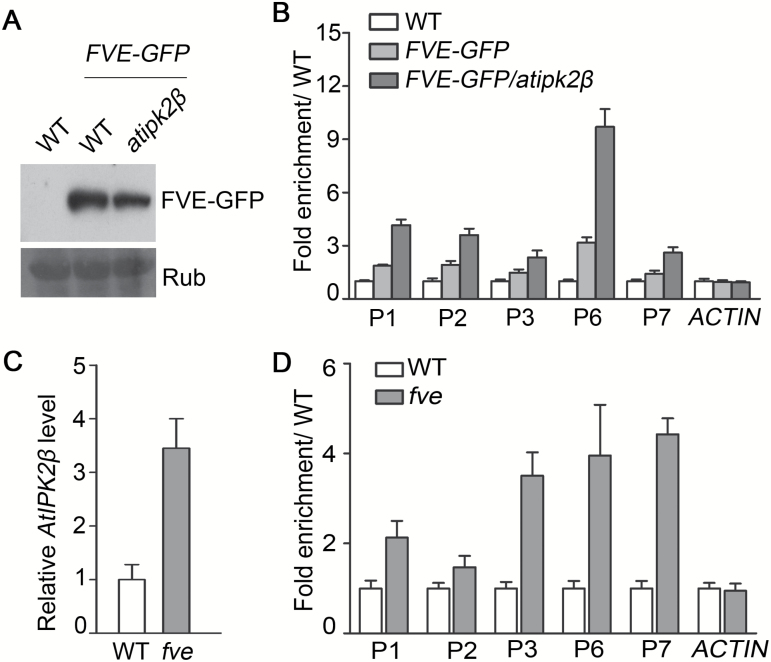
AtIPK2β represses the accumulation of FVE on *FLC* chromatin. (A) Relative levels of FVE-GFP fusion protein in *FVE-GFP* and *FVE-GFP/atipk2β* transgenic plants. Immunodetection of FVE-GFP protein was performed as described in Fig. 4B. (B) Enrichment level of FVE on *FLC* chromatin in the *atipk2β* mutant. ChIP assays were performed as described in Fig. 4C. Transgenic plants overexpressing *FVE-GFP* in the wild-type (WT) and the *atipk2β* mutant background grown under long-day (LD) conditions for 15 days were harvested for ChIP-qPCR assays. Sequence regions examined in ChIP-qPCR assays were the same as those described in Fig. 4A. Fold enrichment levels of *FVE-GFP* in different genetic backgrounds over the control line (WT) are shown as the means±SD of biological duplicates. (C) Relative expression level of *AtIPK2β* in the *fve* mutant. The relative transcript level of *AtIPK2β* was examined by RT-qPCR as described in Fig. 2. Data are presented as the means±SD of two independent repeats. (D) Enrichment level of AtIPK2β on *FLC* in the *fve* mutant. ChIP-qPCR assays were performed using anti-AtIPK2β antibody as described in Fig. 4E. 15-day-old plants grown under LD conditions were harvested for chromatin extraction. Fold enrichment levels of AtIPK2β on *FLC* in the *fve* mutant over the control line (WT) are shown as the means±SD of biological duplicates.

FVE binds to the *FLC* locus by forming protein complexes with chromatin modifiers (Jeon and Kim, 2011; Pazhouhandeh *et al.*, 2011). As AtIPK2β binds to *FLC* chromatin and interacts with FVE, it is presumed that AtIPK2β affects the accumulation of FVE at the *FLC* locus. To confirm this hypothesis, we performed ChIP-qPCR assays using transgenic Arabidopsis overexpressing a *FVE-GFP* fusion construct in the WT and *atipk2β* mutant backgrounds. The transgenic lines were identified and used in the analysis. Similar levels of FVE-GFP protein were identified to be expressed in both the *atipk2β* mutant and WT background (Fig. 6A). The sequence regions examined in the ChIP-qPCR assays were identical to those described in Fig. 4A, and WT was used as a negative control. This experiment confirmed that FVE-GFP accumulates on *FLC* chromatin in both *FVE-GFP* and *FVE-GFP/atipk2β* transgenic plants (Fig. 6B), which supports the reports from previous studies that FVE is enriched at the *FLC* locus (Jeon and Kim, 2011; Pazhouhandeh *et al.*, 2011). Additionally, ChIP-qPCR assays revealed that knockout of *AtIPK2β* results in a greater accumulation of FVE-GFP on *FLC* chromatin in *FVE-GFP/atipk2β* plants compared with that in *FVE-GFP* transgenic plants. We found that AtIPK2β is enriched in sequence regions P1, P2, P6, and P7 of *FLC* in WT (Fig. 4E), and ChIP-qPCR results demonstrated that the enrichment level of the FVE-GFP fusion in the *FVE-GFP* overexpressing line was reduced by more than 50% in these four regions compared to that in *FVE-GFP/atipk2β* transgenic plants (Fig. 6B), suggesting that AtIPK2β represses the accumulation of FVE on *FLC* chromatin.

Next, we examined whether the accumulation of AtIPK2β at the *FLC* locus is affected by FVE. A T-DNA insertion line of *FVE* (SALK_013789) was identified (Supplementary Fig. S3A, B) and used in the analysis. As the mutant line has T-DNA inserted in the first intron of *FVE*, it still expresses a small amount of *FVE* (Supplementary Fig. S3B). Similar to the previously reported *fve* mutants (Gu *et al.*, 2011; Jeon and Kim, 2011; Pazhouhandeh *et al.*, 2011), this T-DNA insertion line exhibits a significantly late-flowering phenotype (Supplementary Fig. S3C). Gene expression analysis showed that the transcription of *AtIPK2*β is promoted in the *fve* mutant (Fig. 6C), and ChIP-qPCR assays demonstrated that the enrichment level of AtIPK2β at the *FLC* locus in the mutant is consistently increased (Fig. 6D). These data showed that FVE negatively regulates the enrichment of AtIPK2β at *FLC*, indicating a dynamic regulatory mechanism of *FLC* expression effected by FVE and AtIPK2β.

### AtIPK2β functions in FVE-mediated transcriptional regulation of *FLC*

**Fig. 7.**
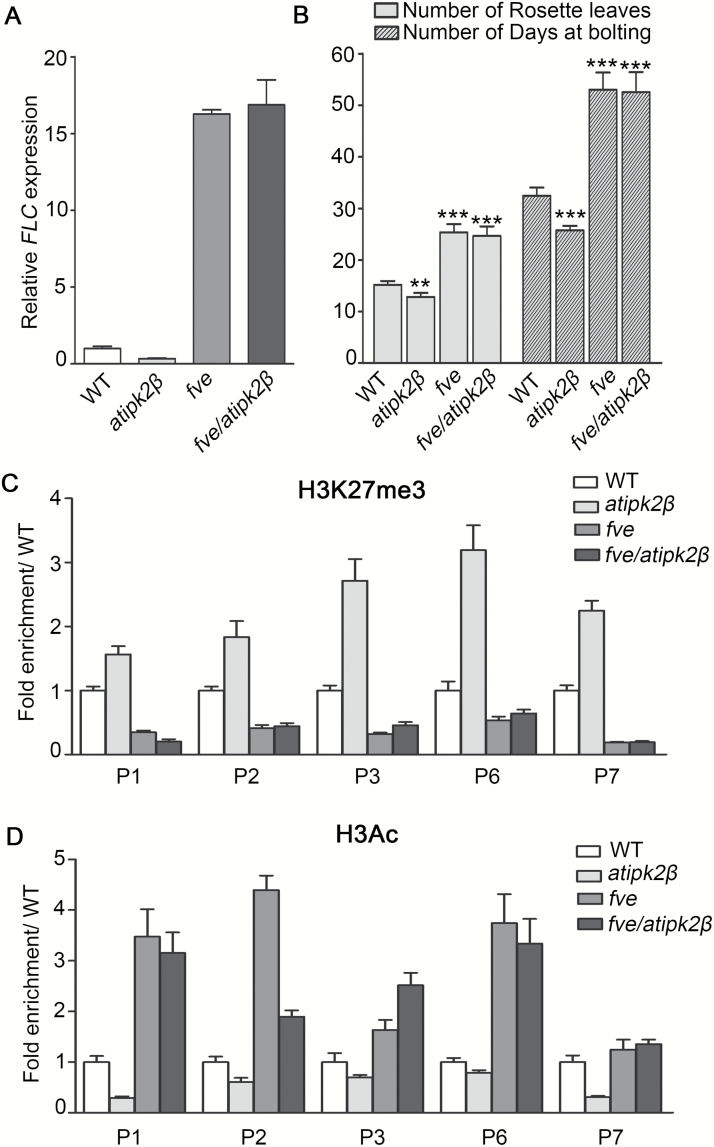
AtIPK2β regulation of *FLC* transcription requires FVE. (A) Expression analysis of *FLC* in wild-type (WT), *atipk2β*, *fve*, and *fve*/*atipk2β* plants. Relative transcript levels of *FLC* in 10-day-old WT and mutant plants grown under long-day (LD) conditions were determined by qPCR, as described in Fig. 2. Data are the means±SD of three biological replicates. (B) Flowering phenotype in WT, *atipk2β*, *fve*, and *fve*/*atipk2β* plants, expressed as the number of rosette leaves at flowering and the number of days to bolting. For each line, 10–15 plants were scored; significant differences (**P<0.01, ***P<0.001) compared to WT were determined by one-way ANOVA with Tukey’s multiple comparison test. (C) Analysis of enrichment levels of the H3K27me3 mark at the *FLC* locus in WT, *atipk2β*, *fve*, and *fve*/*atipk2β*. ChIP-qPCR assays were conducted using H3K27me3 antibody. The amount of DNA precipitates after ChIP was quantified and normalized to *FUSCA*. Data from two independent experiments were averaged and are presented as means±SD; fold enrichments of the H3K27me3 mark on *FLC* in the mutants relative to WT are shown. (D) Analysis of enrichment levels of the H3Ac mark at the *FLC* locus in WT, *atipk2β*, *fve*, and *fve*/*atipk2β*. An anti-acetyl-H3 antibody was used in ChIP-qPCR assays. Chromatin was prepared from 10-day-old WT and mutant plants grown under LD conditions. Sequence regions on *FLC* chromatin examined in ChIP-qPCR assays were identical to those described in Fig. 4A. *ACTIN* was used for normalizing the quantified DNA fragments. Fold enrichments of the H3Ac mark on *FLC* in the mutants relative to WT are shown.

FVE blocks transcription of *FLC* by mediating histone modifications such as H3K27 trimethylation (H3K27me3) and histone H3 deacetylation of *FLC* chromatin (Ausín *et al.*, 2004; Pazhouhandeh *et al.*, 2011; Yu *et al.*, 2016). We have found that AtIPK2β interacts with FVE and represses the accumulation of FVE on *FLC* chromatin, indicating a functional link between AtIPK2β and FVE in the transcriptional regulation of *FLC*. To study whether AtIPK2β regulates *FLC* expression with FVE, we generated the *fve/atipk2β* double mutant and examined the expression level of *FLC* in WT, *atipk2β*, *fve*, and *fve/atipk2β* plants. Fig. 7A shows that the expression level of *FLC* in the double mutant was similar to that in the *fve* mutant, and higher than that in the *atipk2β* mutant. Both the *fve/atipk2β* double mutant and the *fve* mutant consistently exhibited late-flowering phenotypes compared with WT plants (Fig. 7B). These data showed that FVE is required for AtIPK2β regulation of *FLC* expression.

We further determined whether AtIPK2β regulates *FLC* transcription by involvement in FVE-mediated chromatin modification of *FLC*. Consistent with the reduced expression level of *FLC*, ChIP-qPCR assays showed that deposition of the repressive H3K27me3 mark is increased on *FLC* chromatin in the *atipk2β* mutant (Fig. 7C), and enrichment of the active H3Ac mark is decreased at the same locus, compared to that in WT plants (Fig. 7D). In contrast, both *fve/atipk2β* and *fve* mutant plants showed lower H3K27me3 and higher H3Ac levels on *FLC* compared with WT, consistent with the increased level of *FLC* transcription in the mutants. Taken together, these results suggest that AtIPK2β promotes *FLC* expression by blocking FVE-mediated chromatin silencing of *FLC*.

### AtIPK2β associates with HDA6 and attenuates its accumulation on *FLC* chromatin

Previous studies have revealed that FVE interacts with HDA6 to regulate histone modification at the *FLC* locus and mediates chromatin silencing of the target genes (Gu *et al.*, 2011; Yu *et al.*, 2016). HDA6 is one of the Class I HDACs identified in Arabidopsis. It is involved in diverse signaling pathways that interact with different proteins to mediate histone deacetylation and DNA methylation, leading to transcriptional silencing of the target genes (Pandey *et al.*, 2002; Liu *et al.*, 2014). In this study, we have confirmed that AtIPK2β interacts with FVE and represses the accumulation of FVE on *FLC* chromatin. As HDA6 is an interaction partner of FVE, we presumed that AtIPK2β also affects the enrichment level of HDA6 at the *FLC* locus.

**Fig. 8.**
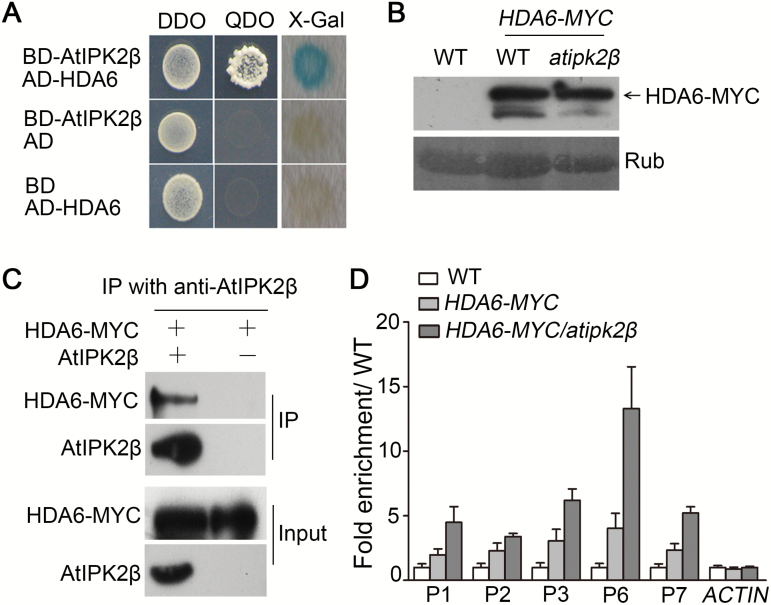
AtIPK2β interacts with HDA6 and attenuates the accumulation of HDA6 on *FLC* chromatin. (A) AtIPK2β interacts with HDA6 in yeast. Yeast two-hybrid assays were performed as described in Fig. 5A. (B) Relative levels of HDA6-MYC fusion protein in *HDA6-MYC* and *HDA6-MYC*/*atipk2β* transgenic plants. Immunodetection of HDA6-MYC was conducted as described in Fig. 4A using anti-MYC antibody. The arrow indicates the band of HDA6-MYC fusion protein. (C) *In vivo* interaction between AtIPK2β and HDA6. Total proteins were extracted from 3-week-old *HDA6-MYC* transgenic plants in the indicated genetic backgrounds and Co-IP assays were conducted as described in Fig. 5C. HDA6-MYC was detected immunologically with anti-MYC antibody. (D) Analysis of HDA6 enrichment on *FLC* chromatin in *HDA6-MYC* and *HDA6-MYC/atipk2β* transgenic plants. Chromatin was extracted from 15-day-old plants grown under long-day conditions. ChIP-qPCR assays were conducted using anti-MYC antibody. Sequence regions examined are identical to those described in Fig. 4A. Data shown are the means±SD of two independent experiments. (This figure is available in colour at *JXB* online.)

We found that HDA6 interacts with AtIPK2β directly in yeast cells (Fig. 8A). *In vivo* Co-IP assays were performed to confirm the interaction by using transgenic plants overexpressing a HDA6-MYC fusion protein in the WT and *atipk2β* mutant backgrounds. Anti-AtIPK2β antibody was applied for immunodetection of AtIPK2β protein in the precipitates. Transgenic plants overexpressing *HDA6-MYC* in the WT background were described previously (Liu *et al.*, 2012); *HDA6-MYC/atipk2β* transgenic plants were generated by crossing the *HDA6-MYC* line with the *atipk2β* mutant and used as a negative control in the Co-IP assays. Similar levels of HDA6-MYC fusion protein were detected in both *HDA6-MYC* and *HDA6-MYC/atipk2β* overexpressing plants (Fig. 8B). The Co-IP assays showed that a band of HDA6-MYC fusion protein was detected in the co-immunoprecipitated proteins from *HDA6-MYC*, but no such band was detected in precipitates from *HDA6-MYC/atipk2β* plants, demonstrating the interaction between AtIPK2β and HDA6 *in vivo* (Fig. 8C).

We further analyzed the effect of AtIPK2β on the accumulation of HDA6 at the *FLC* locus. ChIP-qPCR assays showed that HDA6-MYC is enriched in the examined regions on *FLC* chromatin in both of the transgenic lines overexpressing *HDA6-MYC* in the WT and *atipk2β* background; the enrichment level of HDA6-MYC in *HDA6-MYC/atipk2β* was higher than that in *HDA6-MYC* (Fig. 8D), suggesting that the expression of *AtIPK2β* in *HDA6-MYC* transgenic plants reduces the enrichment level of HDA6 at the *FLC* locus These results indicated that the interaction between AtIPK2β and HDA6 attenuates HDA6 accumulation at the *FLC* locus.

## Discussion

In this study, we report that AtIPK2β promotes *FLC* expression by blocking FVE-mediated chromatin silencing of *FLC*, delaying flowering time. We reveal that AtIPK2β regulates *FLC* expression by enriching at the *FLC* locus. Furthermore, we demonstrate that AtIPK2β blocks FVE-mediated chromatin silencing of *FLC* by interacting with FVE and repressing the accumulation of FVE on *FLC* chromatin. AtIPK2β also interacts with HDA6, an interaction partner of FVE, and represses the accumulation of HDA6 on *FLC* chromatin. This work provides a possible molecular mechanism by which AtIPK2β functions in the transcriptional regulation of *FLC* and flowering time control.

We show that *AtIPK2β* mediates activation of *FLC* transcription (Fig. 2A). Previous studies of *AtIPK2β* indicated its potential function in transcriptional regulation in plants. Although there is no conserved nuclear localization domain found in the protein sequence, localization analysis revealed that AtIPK2β enriches in the nucleus (Xia *et al.*, 2003). Previous investigations showed that overexpressing *AtIPK2β* in Arabidopsis affects the transcription of genes in the auxin signaling pathway, and ectopic expression of *AtIPK2β* in tobacco activates the stress-responsive genes (Zhang *et al.*, 2007; Yang *et al.*, 2008). It has been reported that yeast IPMK (also known as IPK2), which is homologous to AtIPK2β, activates target genes by interacting with a MADS-box transcription factor, Mcm1, and stabilizes the Mcm1-ArgR complex (El Bakkoury *et al.*, 2000; Bosch and Saiardi, 2012). In mammalian cells, IPMK functions in transcriptional activation by interacting with transcription factors and regulators (Malabanan and Blind, 2016). We propose that the function of IPMK in transcriptional regulation is conserved in plants, yeast, and mammals.

We performed ChIP-qPCR assays and revealed that AtIPK2β binds to *FLC* chromatin (Fig. 4C, E). As there is no conserved DNA-binding motif found in the protein structure (Xia and Yang, 2005; Endo-Streeter *et al.*, 2012), it is unlikely that AtIPK2β binds to DNA directly. We noticed that the enrichment level of AtIPK2β on *FLC* chromatin is much higher in the P6 and P7 sequence regions (Fig. 4E), indicating that AtIPK2β enriches in these specific regions, probably through interacting with DNA-binding proteins.

We show in this study that AtIPK2β delays flowering time through FVE-mediated transcriptional regulation of *FLC*. We demonstrated that AtIPK2β interacts with FVE (Fig. 5), which belongs to the conserved MSI1-like protein family that mediates chromatin assembly and histone modification by interacting with histones directly and indirectly (Hennig *et al.*, 2005; Derkacheva *et al.*, 2013). As a positive regulator of floral transition in the autonomous pathway, FVE forms a large complex consisting of at least 16 proteins and binds to *FLC* chromatin, resulting in the inhibition of *FLC* expression (Jeon and Kim, 2011). Previous studies have revealed that FVE regulates histone H3 deacetylation, and histone H3K4 and K27 trimethylation (H3K4me3 and H3K27me3) at the *FLC* locus by forming complex with HDA6 and FLD (Gu *et al.*, 2011; Yu *et al.*, 2016). A study reported that FVE interacts with both CUL4-DDB1 and PRC2-like complexes, and binds to *FLC* and *FT* chromatins dependent of CUL4, leading to enhancement of histone H3K27 trimethylation and histone H3 deacetylation at the *FLC* and *FT* loci (Pazhouhandeh *et al.*, 2011). In this study, we reveal a negative regulatory role of AtIPK2β in FVE-mediated transcriptional regulation of *FLC*. We suggest that the interaction between AtIPK2β and FVE represses the accumulation of FVE on *FLC* chromatin and releases the transcription of *FLC* as a result. The functional link between AtIPK2β and FVE explains the reduced *FLC* expression level in the *atipk2β* mutant, and the late-flowering phenotype with higher *FLC* transcript level in *AtIPK2β*-overexpressing plants. Moreover, FVE represses *AtIPK2β* transcription and therefore reduces the enrichment of AtIPK2β at the *FLC* locus (Fig. 6C, D), indicating a dynamic regulation of *FLC* by both AtIPK2β and FVE.

Recent studies have shown that IPMK plays an important role in the regulation of histone modification in mammalian cells. IPMK physically interacts with CREB binding protein (CBP), which is a histone acetyl transferase, and mediates histone acetylation on the target loci (Xu *et al.*, 2013*a*). IPMK is essential for the recruitment of CBP to chromatins, as deletion of IPMK causes incomplete histone H3 and H4 acetylation, which is dependent on CBP. IPMK is also a critical component for enhancing chromatin association of the p53-p300 histone acetyl transferase complex (Xu and Snyder, 2013). Here, we suggest that AtIPK2β interacts with histone deacetylase HDA6 and attenuates the accumulation of HDA6 on *FLC* chromatin (Fig. 8). Since FVE is essential for HDA6 binding to *FLC* chromatin (Jung *et al.*, 2013), we presumed that AtIPK2β reduces the enrichment of HDA6 on *FLC* by repressing the accumulation of FVE at the *FLC* and probably destabilizing the interaction between HDA6 and FVE. Our investigation suggests a distinguishing function of IPMK in the regulation of histone modification in plant and in mammalian cells.

Taken together, our findings provide evidence for a regulatory mechanism by which AtIPK2β functions in flowering time control. AtIPK2β enriches on *FLC* chromatin and represses FVE-mediated histone modification on *FLC* by attenuating the accumulation of FVE and HDA6 at the *FLC* locus, leading to transcriptional activation of *FLC* and delayed flowering. More widely, our study of the functional link between AtIPK2β and FVE-HDA6 will improve understanding of the *FLC*-dependent pathway in flowering time control. How AtIPK2β interacts with FVE and HDA6 to repress the accumulation of FVE and HDA6 on *FLC* chromatin will be the focus of future investigations.

## Supplementary data

Supplementary data are available at *JXB* online.

Fig. S1. Analysis of flowering time in *atipk2β* mutants and complemented lines under the LD condition.

Fig. S2. Identification of FVE as a potential interactor of AtIPK2β by liquid chromatography coupled to tandem mass spectrometry.

Fig. S3. Molecular characterization of the *fve* mutant.

Table S1. Primer sequences used for genotyping PCR and qRT-PCR.

Table S2. Primer sequences used for ChIP-qPCR.

## Supplementary Material

Supplementary Figures S1-S3 Tables S1-S2
