# Supplementary material for: Arabidopsis inositol polyphosphate multikinase delays flowering time through mediating transcriptional activation of FLOWERING LOCUS C
Source: J Exp Bot. 2017 Nov 17;68(21-22):5787–800. doi: 10.1093/jxb/erx397 (PMC5854132; doi:10.1093/jxb/erx397)
Supplement: Supplementary Figures S1-S3 Tables S1-S2 [file erx397_suppl_supplementary_figures_s1_s3_tables_s1_s2.pdf]

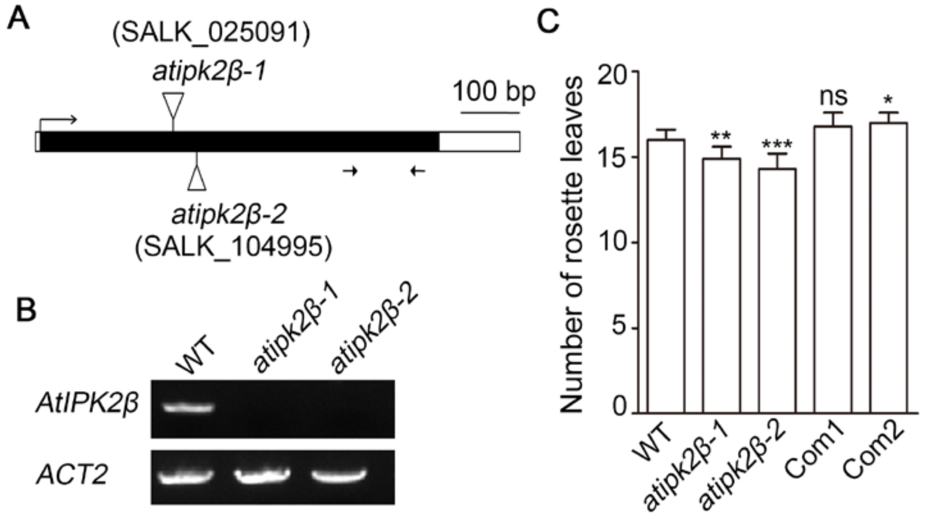

Fig. S1. Analysis of flowering time in *atipk2β* mutants and complemented lines under the LD condition. (A) Structure of *AtIPK2β* gene and T-DNA insertion sites of the two mutant lines. Exons and untranslated regions are presented by black and white boxes respectively. Triangles and arrows indicates T-DNA insertion sites and primers used for RT-PCR. (B) RT-PCR shows the loss of AtIPK2*β* expression in both of the mutant lines. The specific primers for detecting *AtIPK2β* expression is indicated in (A). (C) Flowering time analysis of wild-type (WT), *atipk2β-1,* atipk2*β-2* and two complemented lines of *atipk2β-1* (Com1 and Com2). One-way ANOVA with Tukey's multiple comparison test was used for comparing each value of mutants and complemented lines to WT separately. Asterisk indicates statistically significant difference (**P<0.01, ***P<0.001). ns, not significant.

A FVE (507aa)

MESDEAAAVSPQATTPSGGTGASGPKKRGRKPKTKEDSQTPSSQQQSDVKMKESGKK
TQQSPSVDEKYSQWKGLVPILYDWLANHNLVWPSLSCRWGPQLEQATYKNRQRLYLS
EQTDGSVPNTLVIANCEVVKPRVAAAEHISQFNEEARSPFVKKYKTIIHPGEVNRIRELPQ
NSKIVATHTDSPDVLIWDVETQPNRHAVLGAANSRPDLILTGHQDNAEFALAMCPTEPF
VLSGGKDKSVVLWSIQDHITTIGTDSKSSGSIIKQTGEGTDKNESPTVGPRGVYHGHED
TVEDVAFSPTSAQEFCSVGDDSCLILWDARTGTNPVTKVEKAHDADLHCVDWNPHDDN
LILTGSADNTVRLFDRRKLTANGVGSPIYKFEGHKAAVLCVQWSPDKSSVFGSSAEDGL
LNIWDYDRVSKKSDRAAKSPAGLFFQHAGHRDKVVDFHWNASDPWTIVSVSDDCETTG
GGGTLQIWRMSDLIYRPEEEVVAELEKFKSHVMTCASKP

B

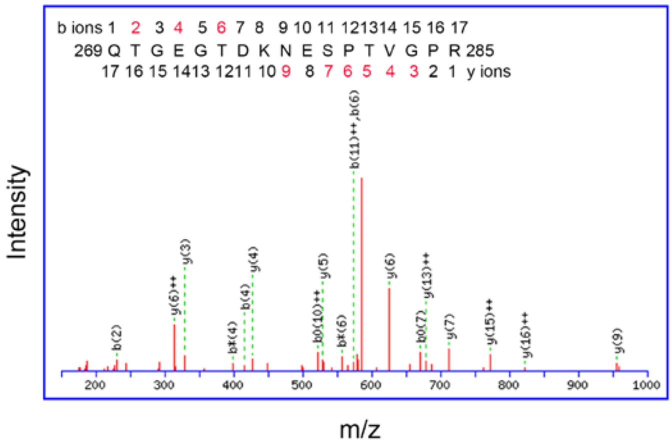

Fig. S2. Identification of FVE as a potential interactor of AtIPK2β by liquid chromatography coupled to tandem mass spectrometry (LC-MS/MS). (A) Protein sequence of FVE and the identified peptide (in red). (B) Corresponding tandem spectra of the peptide detected by LC-MS/MS.

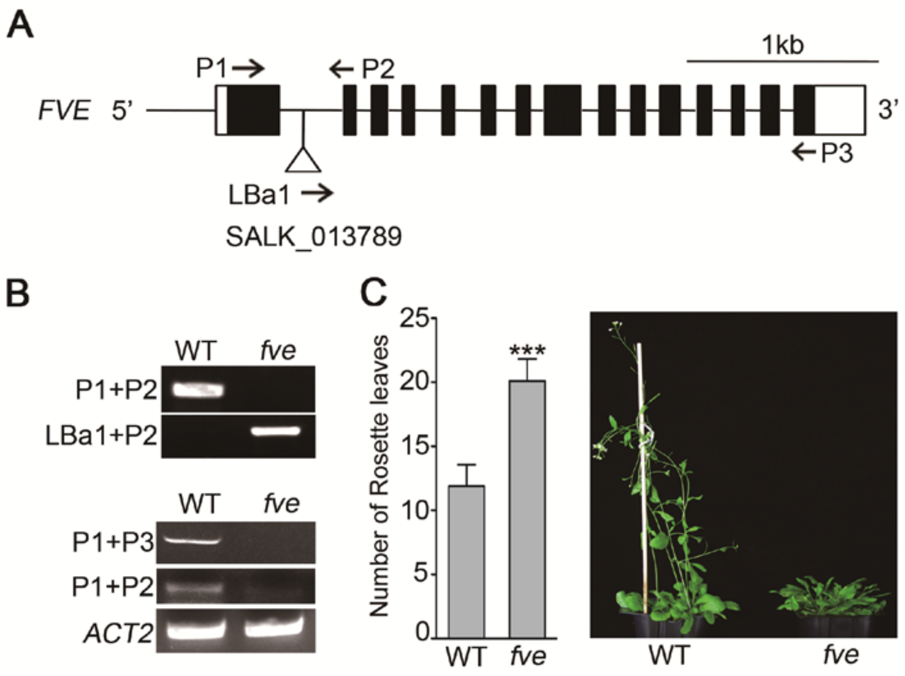

Fig. S3. Molecular characterization of the *fve* mutant. (A) Schematic diagram of *FVE* gene structure. Exons and untranslated regions were represented by black and white boxes, respectively. The T-DNA insertion site was indicated by a triangle. Primers used for identification were marked by arrows. (B) Genotyping PCR of T-DNA insertion (top panel) and expression analysis of *FVE* gene (bottom panel) in *fve* mutant. 10 days old wild-type (WT) and the *fve* mutant grown under the LDs were harvest for RNA extraction and the expression level of *FVE* was analyzed by RT-PCR. *ACT2* was used as an internal control. (C) Flowering phenotypes of the *fve* and wild-type (WT) under the LDs. Rosette leaf number of 10-15 homozygous *fve* mutant and Col0 plants were scored and averaged. The significant difference of the *fve* mutant compared to WT was determined by the t-test (***$P<0.001$). Bars indicate SD.

Table S1. Primer sequences used for genotyping PCR and RT-qPCR

Experiment	Primer Name	Sequence (5'-3')
Genotyping PCR	*fve-P1*	ATGGAGAGCGACGAAGCAGCAGCA
	fve-P2	CGATTCTTGTAGGTTGCTTGC
	fve-P3	GGCTTGGAGGCACAAG
	flc-6-LP	GCTGGACCTAACTAGGGGTGAAC
	flc-6-RP	GTCATTCACGATTTGTTTGATACGATCTG
	LBa1	TGGTTCACGTAGTGGGCCATCG
RT-qPCR	*FT-F*	CTAGCAACCCTCACCTCCGA
	FT-R	TCGTAACACACAATCTCATTGCCAAA
	FLC-F	TGTGGATAGCAAGCTTGTGG
	FLC-R	TAGTCACGGAGAGGGCAGTC
	CO-F	TGCAAGCCAGATGATAACAGTAACAC
	CO-R	TTCCTTGTCTTCCTCTTCTCTCTGTA
	SOC1-F	AGCAGCTCAAGCAAAAGGAG
	SOC1-R	TTGACCAAACTTCGCTTTCA
	AtIPK2β-F	CCGCTGACTCTAACTTGACA
	AtIPK2β-R	TCATCAGAATCGAGCAGGAAT
	ACT2-F	CTTGCACCAAGCAGCATGAA
	ACT2-R	CCGATCCAGACACTGTACTTCCTT
	UBQ10-F	GGCCTTGTATAATCCCTGATGAATAAG
	UBQ10-R	AAAGAGATAACAGGAACGGAAACATAGT

Table S2. Primer sequences used for ChIP-qPCR

Gene	Amplified Region	Sequence (5'-3')
FLC	P1	F: TCAGTTTCACCCACTCCGAG<br>R: AGTAACAAGACGAAAAGAAGTGGG
	P2	F: CAAGCTGATACAAGCATTTCACCAA<br>R: TGTCCACACATATGGCAATAGCTCAA
	P3	F: CCTAATTTGATCCTCAGGTTTGGG<br>R: CCGACGAAGAAAAAGTAGATAGGCAC
	P4	F: GTCATTCACGATTTGTTTGATACGATCTG<br>R: AAGGTAAGAAACAATCAAAGATGGC
	P5	F: TGTAAGCCACATTAATTGGGAA<br>R: GTGTGTAACTGCAAGAGTGGG
	P6	F: GGCTTCCTCATACTTATGGTTATCTG<br>R: CTTGAGGACAAGGTTTTTTCCAG
	P7	F: CCTCTCCGTGACTAGAGCCAAG<br>R: CTTCAACATGAGTTCGGTCTGC
FT	P1	F: GCCTCTACTAGCTCCACCG<br>R: GAAGATAATTACAAAGACACATAC
	P2	F: AATCTTGTCTGCGACTGCGACCTA<br>R: GCATTTTTAAATATTGGACAGGAG
	P3	F: TGATATAATTGTTTCAAGACACTAG<br>R: ACTCAAATACGCAAAATTATCTC
	P4	F: GAGACCCTCTTATAGTAAGCAGAGTTG<br>R: GGGAGTTCAAGTGAAAGAACCAAAGT
	P5	F: CTCAAACATGTTGCTCGAATTATA<br>R: GGTGTGGGCTTTTTTGGGAGAC
	P6	F: GATCTACAATCTCGGCCTTCC<br>R: ATCATCACCGTTCGTTACTCG
ACTIN2	*ACTIN*	F: CGTTTCGCTTTCCTTAGTGTTAGCT<br>R: AGCGAACGGATCTAGAGACTCACCTTG
FUSCA3	*FUSCA*	F: AGTTGGCACGTGGGAAATAG<br>R: GTGGCAAGTGTTGATCATGG
